# Exploring the Co-Structure of Physical Activity and Dietary Patterns in Relation to Emotional Well-Being: A Tanglegram-Based Multivariate Approach

**DOI:** 10.3390/nu17142307

**Published:** 2025-07-13

**Authors:** Jarosław Domaradzki, Małgorzata Renata Słowińska-Lisowska

**Affiliations:** Department of Biological Principles of Physical Activity, Wroclaw University of Health and Sport Sciences, 51-612 Wrocław, Poland; malgorzata.slowinska-lisowska@awf.wroc.pl

**Keywords:** physical activity patterns, dietary patterns, mental health, emotional well-being, young adults

## Abstract

**Background/Objectives**: Psychological distress is common among university students and often co-occurs with unhealthy lifestyle patterns. However, most studies examine physical activity (PA) and dietary intake (DI) in isolation, overlooking how these behaviors interact under stress. This study aimed to identify and compare integrated PA and DI behavior patterns among students with low vs. high psychological distress. **Methods**: A cross-sectional case–control design was used with 209 students (aged 19–21). Questionnaires included the International Physical Activity Questionnaire (IPAQ), Questionnaire of Eating Behavior (QEB), and Depression Anxiety Stress Scales-21 items (DASS-21). Behavioral patterns were assessed using a cophylogenetic approach (tanglegrams, cophenetic statistics), and predictive behaviors were analyzed using stepwise logistic regression. **Results**: Permutational Multivariate Analysis of Variance (PERMANOVA) revealed significant group differences in PA–DI structure (F = 3.91, R^2^ = 0.0185, *p* = 0.001). Tanglegram and PACo analyses showed tighter PA–DI alignment in high-distress individuals, suggesting more rigid, compensatory behavior profiles. Logistic regression identified vigorous PA (OR = 1.80, 95% CI: 1.33–2.50, *p* < 0.001) and fast food intake (OR = 1.43, 95% CI: 1.05–1.98, *p* = 0.026) as significant distress indicators. Sweets intake showed a non-significant trend (OR = 1.33, *p* = 0.064). **Conclusions**: Students with higher psychological distress exhibit complex lifestyle co-patterns combining risk (e.g., fast food) and compensatory behaviors (e.g., vigorous PA). Health promotion should address PA and DI jointly, and screening for distress should be integrated into student wellness programs.

## 1. Introduction

The relationship between physical activity (PA), dietary intake (DI), and emotional stress in young people is complex and interrelated [1,2,3]. Physical activity contributes to better mental well-being and can help reduce stress in students. Studies show that regular physical exercise is associated with lower stress levels and improved mood among students [2]. This relationship is undoubtedly bidirectional: greater stress and negative emotions can reduce physical activity levels, while engaging in physical activity can reduce stress and improve mood [4]. In a study by Lucini et al., students themselves reported that they cope with stress by increasing their physical activity and are aware of its health benefits [5].

The quality of diet also plays an important role in maintaining mental well-being. Eating unhealthy foods is associated with greater stress, while following proper eating patterns can reduce stress symptoms [1]. In a study by Cedillo et al. (2023) [6] found that dietary irregularities among college students are common and associated with higher BMI, with mental well-being and stress playing a mediating role in these relationships. The study highlights the need for targeted interventions to support the overall health and well-being of students [6]. An interesting issue seems to be the analysis of the interaction between physical activity and diet as important elements in reducing stress in young people. Some authors suggest that increasing physical activity may have a greater impact on body composition than diet alone. Promoting physical activity is very important for maintaining the health of young people [7]. It has also been found that encouraging regular physical exercise and adherence to a Mediterranean diet can improve body satisfaction and reduce the risk of eating disorders [8].

Of particular interest are studies conducted during the COVID-19 lockdown. This period led to a reduction in work-related physical activity and an increase in sedentary time, changes in eating habits, and a deterioration in mental well-being among the Florentine academic community [2]. Over the past few years, there has also been a growing trend of emotional eating among university students. Currently available research has confirmed that emotional eating is primarily associated with female gender, BMI, physical inactivity, depression, anxiety, stress, sleep disorders, and social media abuse [3].

The interplay between PA, DI, and emotional stress has gained increasing attention in health psychology and behavioral science. Numerous studies have highlighted the protective effects of regular PA on mental health, showing that physical activity—particularly moderate to vigorous intensity—can reduce symptoms of anxiety, depression, and stress through physiological (e.g., endorphin release, HPA axis modulation) and psychological mechanisms (e.g., improved self-esteem, distraction from rumination) [9,10,11]. Furthermore, PA is often promoted as a self-regulatory strategy for emotional coping, particularly among young adults navigating transitional life phases such as university studies [12].

Dietary behaviors are also closely linked to emotional well-being. Emotional stress has been associated with both undereating and overeating, depending on individual traits and context [13]. A common stress-related pattern, often referred to as “emotional eating,” involves increased intake of energy-dense, high-sugar, and high-fat foods, such as sweets and fast food [14]. These dietary choices may offer short-term mood regulation but contribute to long-term health risks. Additionally, the bidirectional nature of the diet-mood relationship is underscored by evidence suggesting that poor nutritional quality (e.g., low intake of fruits, vegetables, omega-3 fatty acids) can contribute to increased vulnerability to depression and anxiety [15].

Emerging research suggests that PA and DI behaviors often do not operate independently but rather form complex, compensatory patterns—particularly in individuals under stress. For example, some individuals may offset unhealthy eating habits by increasing physical activity, while others may reduce both PA and diet quality simultaneously during high-stress periods [16,17]. Understanding these integrated behavior patterns is critical for developing targeted health promotion strategies that address the full complexity of lifestyle and emotional regulation among university students.

A gap in our knowledge about the effects of PA and DI on emotional stress is their interaction. For older adolescents/young adults, combinations of PA and diet given their hormone levels and physiological development may have a range of effects depending on PA level, dietary habits, and other factors that can affect well-being. Therefore, the main objective of this study was to identify and compare complex patterns of lifestyle-related behaviors—defined by the integration of PA and DI variables—among university students experiencing varying levels of psychological stress. Accordingly, our study focuses on emotional well-being rather than clinical diagnosis. Specifically, we aimed to (1) examine overall multivariate differences in PA–DI behavioral configurations between low-distress (LD) and high-distress (HD) students using Permutational Multivariate Analysis of Variance (PERMANOVA) analysis; (2) construct and analyze the structural relationships between PA and DI variables in each group through co-phylogenetic analysis and tanglegram-based comparisons, evaluating the strength and coherence of these inter-domain associations; (3) visualize and compare distinct within-domain behavioral linkages (i.e., PA-to-PA and DI-to-DI) using two-face tanglegrams, highlighting patterns that are unique or divergent between low-distress (LD) and high-distress (HD) groups and to identify key behavior clusters that contribute most to group differentiation using dendrogram difference analysis, focusing on which specific PA and DI behaviors are structurally most dissimilar across distress levels; and (4) evaluate the predictive value of the identified behaviors using multiple logistic regression, estimating odds ratios (ORs) to quantify the likelihood of being in the HD group (whether the presence of particular behaviors meaningfully increases the odds of distress, thus offering potential behavioral signatures or markers that may help identify vulnerable individuals).

## 2. Materials and Methods

### 2.1. Study Design

A cross-sectional, non-random sample of university students participated in the study. Study recruitment and methodology are detailed elsewhere [18]. This analysis used a group-comparison approach aligned with a case–control framework, suitable for examining behavioral and psychological differences between individuals with and without a given mental health characteristic. It enabled structured assessment of lifestyle factors linked to varying distress levels.

### 2.2. Ethics

Ethical approval was obtained from the Senate Research Ethics Committee of Wroclaw University of Health and Sport Sciences (No. 13/2022). Participants gave informed e-consent after receiving full study information.

### 2.3. Sample Size

Sample size was determined using standard heuristics for exploratory multivariate analysis. To support clustering of 16 dietary and 4 physical activity variables, and logistic regression stratified by sex, a target of ≥240 participants was set—ensuring ≥30 cases per cluster or 10–20 cases per predictor for stable odds estimation [19,20,21].

### 2.4. Participants

**Figure 1:**
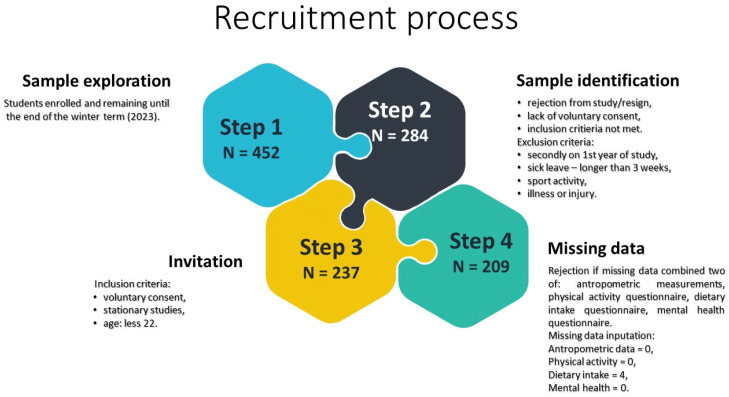
Flowchart of the recruitment process.

A total of 237 healthy first-year students (44% male) from physical education and physiotherapy programs at Wroclaw University of Health and Sport Sciences (2023) were initially recruited. To confirm that participants were healthy, a brief structured interview was conducted prior to enrollment. Students reported whether they regularly attended physical education classes and were medically cleared to participate. They were also asked if they had experienced any illness lasting more than seven days or sustained any injury within the past two months. Only those with no recent illness or injury and with medical clearance were included in the study. Participants were recruited as volunteers in response to an invitation presented during regular school activities. They did not receive any course credit or financial compensation for their participation. After excluding 28 participants due to missing data across multiple domains, the final analytical sample included 209 students (91 males and 118 females). Figure 1 presents the sampling flow in detail. Gender distribution reflected that of the target population. Prior data [21] showed high physical activity levels, with ≥3000 MET-min/week reported by 81% of males and 76% of females. Figure 1 presents the sampling flow and final analytical sample.

Eligibility required attendance in on-campus courses, age under 22, and informed consent. Exclusion criteria included university-level athletic training, medical leave (>3 weeks), or current illness/injury. Of 237 eligible students, 28 were excluded for missing data across multiple domains. Minor gaps in dietary intake data (*n* = 4) were addressed via the imputation method described below.

The final sample included 209 students: 91 males and 118 females. Male participants had a mean height of 183.64 cm (SD = 7.09), weight of 79.17 kg (SD = 10.03), and BMI of 23.56 kg/m^2^ (SD = 2.39), indicating a normal weight range. Female participants averaged 168.72 cm in height (SD = 5.30), 61.13 kg in weight (SD = 8.38), and had a BMI of 21.59 kg/m^2^ (SD = 2.74), also within the normal range.

A total of 28 students were excluded from the initial sample. Reasons for exclusion included incomplete dietary and psychological questionnaires, missing anthropometric data, and refusal to participate. The final sample consisted of students who met all inclusion criteria and provided complete data for analysis.

Despite the reduced final sample (*n* = 209), we were able to maintain adequate statistical stability for the exploratory goals of the study.

### 2.5. Data Collection

Data were collected through the Family Lifestyle Patterns project (FAST-PAT23), which assessed physical activity, dietary habits, health-related attitudes, and socio-economic factors. Closed-ended questionnaires were administered via Google Forms immediately following a lecture (Human Anatomy) taught by one of the authors. Recruitment, data collection, and entry were conducted by the same author. Anthropometric measurements were taken over four consecutive weeks in March 2023, following a prearranged schedule.

To reduce individual-level variance attributable to sex or other demographic factors, all continuous variables were z-transformed (standardized) within the entire sample before analysis. This allowed us to investigate relative behavioral and psychological patterns across the whole cohort rather than focus on absolute group differences. This technique is commonly used in mixed-group physiological, anthropological, and behavioral studies to enable pattern-level comparisons across heterogeneous populations.

### 2.6. Anthropometric and Body Composition Measurements

Anthropometric measurements were performed in the Biokinetics Research Laboratory at Wroclaw University of Health and Sport Sciences, part of the Central Research Laboratory. The facility is certified under the Quality Management System standards PN-EN ISO 9001:2009 (Reg. No. PW-48606-10E) and PN-EN ISO 9001:2015 (Reg. No. PW-15105-22X). Body height was measured twice to the nearest 0.1 cm using a GPM anthropometer. Body weight and composition were assessed with the InBody230 analyzer. Body mass index (BMI) was subsequently calculated using formula: BMI = body weight [kg]/body height^2^ [m^2^].

### 2.7. Questionnaire Measurements

#### 2.7.1. Physical Activity Questionnaire

Students’ physical activity levels were assessed using the Polish version of the IPAQ-Long Form, administered online via Google Forms following standardized guidelines. The questionnaire includes 11 items covering four domains—work/study, transport, domestic/gardening, and leisure—and one item on sitting time. Responses were converted to MET-min/week, with analyses including total PA, domain-specific activity, and sitting duration.

#### 2.7.2. Dietary Intake Questionnaire

Dietary intake over the prior 12 months was assessed using the self-administered Questionnaire of Eating and Behaviors (QEB) [22], based on a food frequency approach. The tool has shown good reliability (Fleiss’ kappa = 0.64−0.84) [23]. Of the original 21 items, the validated 16-item core set was used, as recommended by the developers [23].

Food group consumption was reported on a six-point frequency scale, from “never” to “several times per day.” Responses were converted to average daily intake values: never = 0, 1–3/month = 0.06, once/week = 0.14, several/week = 0.5, daily = 1, and several/day = 2.

Dietary quality was assessed using two composite indices: (1) a pro-healthy index including wholegrain bread, milk, fermented dairy, curd cheese, fish, legumes, fruits, and vegetables, and (2) an unhealthy index comprising fast food, fried foods, cream cheese, sweets, canned meat/fish, sweetened beverages, energy drinks, and alcohol.

#### 2.7.3. Depression, Anxiety, and Stress Questionnaire

The Depression, Anxiety, and Stress Scale-21 Items (DASS-21) is a validated self-report tool measuring three related emotional states: depression, anxiety, and stress [24]. It is a shortened version of the original 42-item scale, widely used in both clinical and research settings.

The questionnaire comprises 21 items divided evenly into three subscales [25]:

- Depression (7 items): low mood, hopelessness, low self-esteem, and loss of motivation or interest;
- Anxiety (7 items): physiological arousal, situational fear, and panic-like symptoms;
- Stress (7 items): chronic tension, irritability, and difficulty relaxing.

Participants were classified into high or low psychological distress groups based on established DASS-21 thresholds. The high distress group included individuals who either

- Had a total DASS-21 score > 60;
- Scored in the severe/extremely severe range on at least one subscale—Depression > 21, Anxiety > 15, or Stress > 26 [26].

All others were categorized as low distress.

Although the DASS-21 is primarily a screening measure and not a diagnostic tool, we selected it due to its brevity, strong psychometric properties, and suitability for adolescent school-based research. Its use enables a multidimensional assessment of emotional well-being (across depression, anxiety, and stress) within a limited timeframe, which was crucial given the large sample and school setting. We acknowledge that DASS-21 does not cover all behavioral or functional aspects of stress (e.g., sleep issues, academic performance, or eating problems beyond sugar intake). However, we also assessed selected health-related behaviors such as physical activity and dietary habits, including added sugar consumption, which are commonly linked with emotional distress. These complementary data provide partial behavioral context for interpreting DASS-21 scores. The limitations of relying on a single psychological instrument are further discussed in the Discussion section.

### 2.8. Handling and Imputation of Missing Data

No missing data were found in anthropometric, body composition, or physical activity datasets. However, four participants had incomplete responses in the dietary intake questionnaire (QEB). Since principal component analysis (PCA) requires complete data, multiple imputation was applied. The missingness mechanism was identified as missing completely at random (MCAR) [27,28], indicating no systematic bias. Imputation was conducted in R (RStudio v.2024.11.0) using the mice package (v.3.14.0; RStudio, PBC, Boston, MA, USA; accessed 15 November 2024).

### 2.9. Data Analysis

Responses from the DASS-21, IPAQ, and QEB questionnaires were transformed using the Yeo–Johnson power transformation to approximate normal distributions [29]. To ensure comparability across differently scaled variables and to combine male and female data, all values were standardized (mean = 0, SD = 1). Normality of the transformed and standardized data was confirmed using the Shapiro–Wilk test, allowing for the use of parametric descriptive statistics: mean, 95% confidence interval (CI), and standard deviation. Group differences in anthropometric measures, physical activity, dietary intake, and emotional well-being (between low-distress [LD] and high-distress [HD] groups) were analyzed using independent Student’s *t*-tests.

Initially, Permutational Multivariate Analysis of Variance (PERMANOVA) was applied to assess whether multivariate lifestyle behavior patterns—integrating physical activity (PA) and dietary intake (DI)—differed between low-distress (LD) and high-distress (HD) groups. This method was chosen for its robustness to violations of multivariate normality and its ability to evaluate group-level structure using Euclidean distances. Next, relationships between PA and DI patterns were explored within and between distress groups using tanglegrams and cophenetic statistics, derived from a cophylogenetic framework. Within each group (LD and HD), tanglegrams were generated to visualize clustering of PA and DI variables based on Euclidean distances and Ward’s linkage method. Procrustes and Procrustean Approach to Cophylogeny (PACo) tests were then performed to formally assess structural similarity between PA and DI configurations in each group. Tanglegrams and cophenetic statistics have also found applications in the social sciences focused on human behavior, including sociology, psychology, and education [30,31]. Tanglegrams are particularly effective for visualizing hierarchical relationships between sets of traits, opinions, or behaviors—especially when comparing patterns such as personal values versus lifestyle habits, coping strategies, or social preferences. Their strength lies in the intuitive representation of structural relationships between two clustering trees, allowing for deeper insights into the degree of alignment or divergence between behavioral domains. To identify items that uniquely distinguished between groups, functions from the dendextend package in R were used, specifically, dend_diff and all.equal. The dend_diff function visualizes differences by displaying two dendrograms side by side, highlighting unique edges in red and shared edges in black, aiding visual interpretation. The all.equal function provides a global comparison by listing edges unique to each tree, helping to isolate items contributing to group-level differences. Finally, stepwise multiple logistic regression was used to assess prediction power of the identified lifestyle behaviors specifically distinguished between low- and high-distressed participants and to assess the odds to present such behaviors being high distressed.

The significance level for all statistical tests was set at α = 0.05. All analyses were performed using RStudio (v.2024.11.0) and Statistica 13.0 (StatSoft, Cracow, Poland, 2018).

## 3. Results

### 3.1. Basic Descriptive Characteristic of the Low and High Distressed Groups

To enable meaningful analysis across the entire study population—including both men and women—the data were normalized and transformed. This step was necessary due to inherent sex-based differences in physical activity levels, dietary intake, and psychological responses, which could otherwise obscure genuine behavioral patterns when analyzed collectively. By applying normalization and appropriate transformation procedures (e.g., z-score standardization or Yeo–Johnson transformation), we ensured that all variables were placed on a comparable scale, reducing the influence of distributional differences related to sex.

Analyzing the combined group is theoretically justified, as it allows for the identification of generalizable behavioral structures and interaction patterns that transcend gender. This approach is particularly relevant in public health and educational contexts, where interventions are often designed and implemented at the population level rather than separately by sex. Moreover, integrating data from men and women increases statistical power and improves the robustness of multivariate pattern detection methods, such as clustering or co-structure analysis. Therefore, normalization was a crucial step to uncover shared PA–DI–distress dynamics across the full sample, without being confounded by baseline sex-related disparities.

Based on the DASS-21 classification criteria, a total of 130 students were categorized as having low psychological distress (males: 67, females: 63), and 79 students were classified as having high psychological distress (males: 24, females: 55). These groupings were used in subsequent analyses comparing psychological distress levels across physical activity and dietary behavior variables. Significant differences were found between LD and HD groups across all DASS-21 components (*p* < 0.001). LD participants reported more moderate, transport-related, and domestic/gardening activity, while HD individuals engaged more in vigorous and leisure-time activity. The HD group also consumed more fast food, sweets, canned meals, and sweetened beverages (all *p* < 0.05), indicating a shift toward processed dietary patterns among those with higher distress. Details are presented in Table 1.

### 3.2. Congruence Between PAPs and DPs in the Low and High Distressed Groups

PERMANOVA based on Euclidean distances revealed a significant difference in the overall multivariate structure of physical activity and dietary intake between LD and HD groups (F = 3.91, R^2^ = 0.0185, *p* = 0.001). Although the effect size was modest, the findings indicate subtle but consistent lifestyle behavior differences linked to emotional distress.

Tanglegram visualizations of physical activity (PA) and dietary intake (DI) revealed distinct behavioral pattern structures between the LD and HD groups (Figure 1).

In the LD group, PA variables formed three clusters: (1) moderate activity, domestic and gardening, transport, walking, and working; (2) vigorous and leisure-time activity; and (3) sitting time. The first cluster was linked to fried meals and sweetened beverages. Working, walking, and transport were associated with curd and yellow cheese intake, while vigorous leisure-time activity correlated with higher consumption of sweets and legumes. Increased sitting time was most often associated with fermented milk intake.

In the HD group, four PA clusters emerged: (1) transport and walking; (2) moderate activity with working and domestic gardening; (3) vigorous leisure-time activity (as in LD); and (4) sitting time, forming its own cluster. The first cluster was linked to healthier dietary habits, including higher fruit and vegetable intake. In contrast, moderate activity related to work was associated with fried meals and sweetened beverages, while domestic gardening correlated with curd cheese. Vigorous leisure activity was linked to canned meals and yellow cheese. Higher sitting time was associated with increased fast food consumption.

Cross-domain links between PA and DI were more thematically structured in the HD group. Vigorous PA often coincided with fast food intake, and moderate PA with fried meals, suggesting more consolidated—but potentially compensatory—lifestyle patterns among individuals with higher emotional distress.

These visual patterns were statistically evaluated using Procrustes analysis, which assessed the alignment between PA and DI behavior profiles within each group. In the LD group, the Procrustes correlation was t_0_ = 0.191 (*p* = 0.750), indicating a weak, non-significant relationship. The HD group showed a slightly higher, yet still non-significant, correlation of t_0_ = 0.282 (*p* = 0.127). These results suggest that PA and DI behaviors function largely independently at the individual level. However, the more coherent pairings in the HD group may reflect a tendency toward tighter behavioral coupling under emotional distress.

Unlike Procrustes, PACo analysis evaluates structural congruence based on internal pairwise relationships. It revealed significant PA–DI alignment in both groups. In the LD group, the sum of squares was 126.36 (*p* = 0.01), indicating non-random structure. The HD group showed an even stronger match, with a lower sum of squares (87.08, *p* = 0.002). These findings suggest that emotionally distressed individuals exhibit more tightly organized PA and DI behavior patterns than would be expected by chance.

These findings support the notion that individuals under emotional strain may rely on more rigid or habit-driven combinations of physical activity and dietary choices, whereas those with lower distress levels may maintain more flexible and independently regulated lifestyle patterns.

### 3.3. Congruence in Patterns of Behavior Between the Low and High Distressed Groups

Similarity between analogous behaviors (PA and DI) was assessed in two stages. First, paired dendrograms for LD and HD groups were visually compared side by side, separately for PA and DI, with matching labels directly connected. Second, this visual assessment was supported by cophenetic analysis to quantify structural similarities.

**Figure 2:**
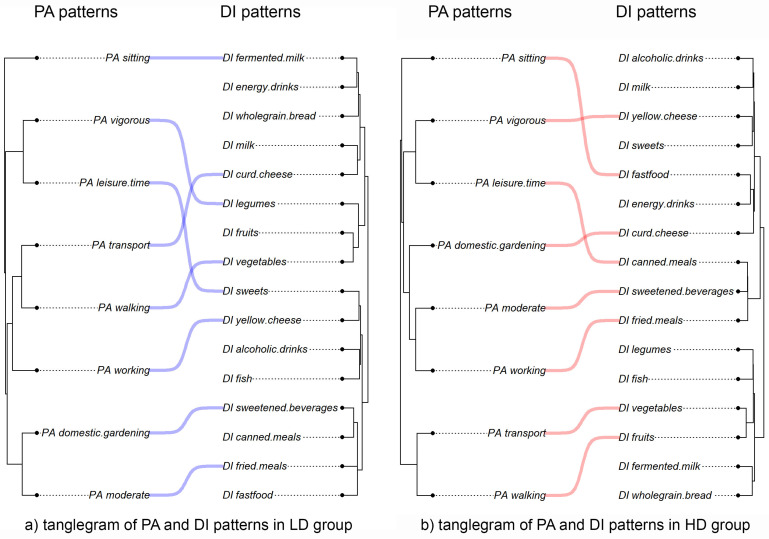
Tanglegrams of physical activity (PA) and dietary intake (DI) behavior patterns in low distress (LD) and high distress (HD) groups. The dendrograms on the left display hierarchical clustering (Euclidean distance, Ward. D2 linkage) of PA variables, while the dendrograms on the right show clustering of DI variables. Curved lines represent optimal one-to-one associations between PA and DI variables based on Euclidean distance (Hungarian matching). Panel (**a**) shows the pattern structure in the LD group, and panel (**b**) shows the structure in the HD group. Differences in cross-domain pairings suggest group-specific behavioral organization, with HD participants showing more tightly structured links between physical activity and dietary intake behaviors.

**Figure 3:**
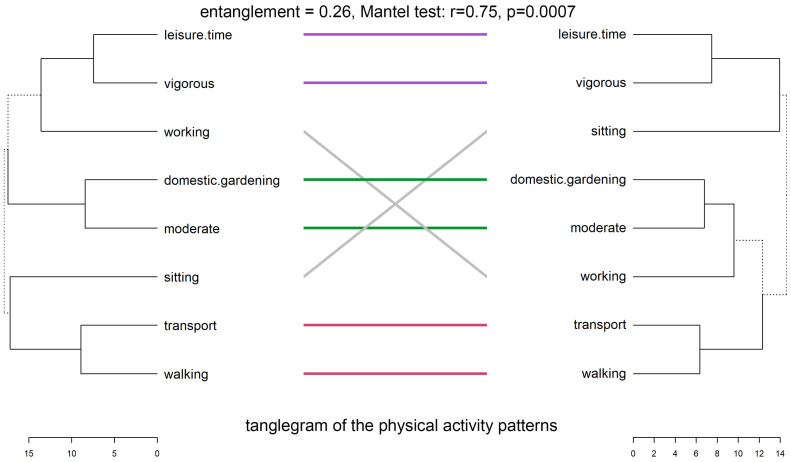
Tanglegrams of the physical activity patterns in LD and HD. Euclidean distances and Ward’s method of linkage were used. Primary dendrograms were untangled. Bottom, horizontal open bars present distances between items in each cluster. Dashed lines indicate the most differences in LD and HD patterns.

Tanglegrams (Figure 2 and Figure 3) illustrated the degree of congruence between LD and HD dendrograms for PA and DI patterns. Trees were aligned using cophenetic correlations, with matching substructures connected by identically colored lines to highlight similarities. To enhance clarity, an untangling algorithm was applied to reduce line crossings and improve the readability of the tanglegrams, resulting in clearer visualizations than the original layouts.

#### 3.3.1. Physical Activity Patterns

Figure 3 shows the tanglegram comparing hierarchical clusterings of physical activity patterns (PAPs) between LD and HD groups. The entanglement value decreased from 0.26 to 0.11 after untangling, indicating a moderate improvement in clarity. Visually, both dendrograms reveal similar behavior groupings, with several subtrees consistently aligned, as highlighted by colored connecting lines.

In both dendrograms, leisure-time and vigorous activity consistently cluster together, as do moderate activity and domestic gardening, reflecting shared behavioral structures. However, differences emerge in the hierarchical positioning of working and sitting, which fall into separate sub-clades across groups—suggesting variations in activity context or co-occurrence patterns.

The cophenetic correlation between the two dendrograms was r = 0.72, indicating moderate structural similarity. This was supported by the Mantel test (r = 0.75, *p* = 0.0007), confirming a strong, significant correlation between distance matrices. The Fowlkes–Mallows Index (FMI) was 0.34, slightly above the null expectation of 0.31, suggesting modest but meaningful alignment in clustering structure.

Descriptive statistics showed comparable average pairwise distances in PAPs: 13.46 ± 2.46 for LD and 10.47 ± 1.99 for HD, indicating similar overall variability within each group.

Overall, these findings indicate moderate congruence in physical activity structures between LD and HD groups, with both overlapping and distinct behavioral clustering patterns.

#### 3.3.2. Dietary Intake Patterns

**Figure 4:**
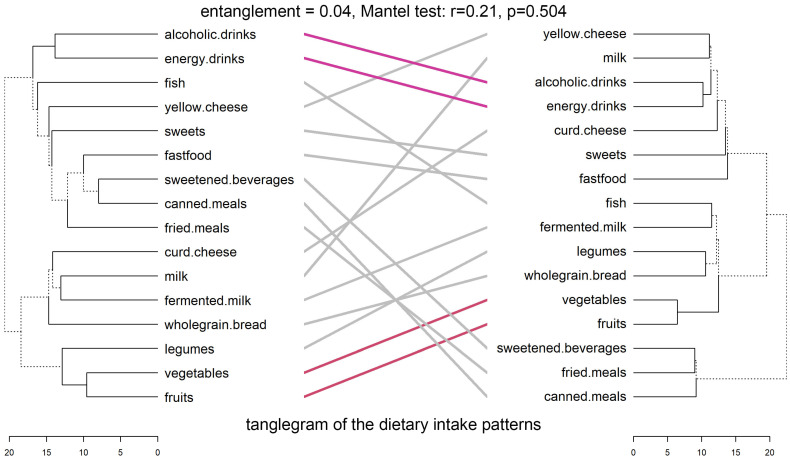
Tanglegrams of the dietary intake patterns in LD and HD. Euclidean distances and Ward’s method of linkage were used. Primary dendrograms were untangled. Bottom, horizontal open bars present distances between items in each cluster. Dashed lines indicate the most differences in LD and HD patterns.

Figure 4 displays the tanglegram comparing dietary intake patterns (DIPs) between LD and HD groups. Initial entanglement was high (0.70), indicating notable misalignment. After untangling, it dropped to 0.04, revealing clearer and more interpretable correspondence between diet-related variables across groups.

Despite some matched variable pairs—highlighted by colored lines—the overall dendrogram structures differ between LD and HD groups. For instance, fermented milk and milk cluster together in both groups, but items like fast food, sweets, and energy drinks appear in different sub-clades, reflecting divergence in dietary patterns linked to emotional distress.

Cophenetic correlation between dendrograms was low (r = 0.21), indicating weak structural similarity. Baker’s Gamma (r = 0.11) also showed low, though non-zero, correspondence. The Mantel test found no significant relationship between distance matrices (r = −0.00009, *p* = 0.504), supporting limited overall similarity. However, the Fowlkes–Mallows Index (FMI = 0.43) exceeded the null expectation (0.35), suggesting modest but non-random overlap in cluster memberships.

Descriptive statistics showed slightly higher mean pairwise distances in the LD group (14.23 ± 1.72) than in the HD group (12.87 ± 1.67), suggesting greater internal variability in dietary behaviors among emotionally healthier individuals.

Overall, the findings suggest that while some pairwise associations are preserved, the clustering of dietary intake behaviors differs meaningfully between LD and HD groups, likely reflecting distinct co-occurrence patterns shaped by emotional distress.

Based on dendrogram topology comparisons and clustering differences identified using all.equal() and dend_diff(), a subset of PA and DI behaviors that shifted structurally between LD and HD groups was selected. These variables were entered into a multiple logistic regression model to assess their predictive value for high distress. Odds ratios (ORs) with 95% confidence intervals were calculated to quantify the associated risk for each behavior.

To identify candidate variables associated with emotional distress, hierarchical dendrograms were compared using the all.equal() function. For physical activity (PA), the LD and HD dendrograms showed a mean relative difference in branch heights of 0.218, indicating moderate divergence in clustering strength. Topological differences were also noted, with unique edges (edge 7 in LD and edge 6 in HD) reflecting localized reorganization of activity patterns across distress levels.

For dietary intake (DI), the mean branch height difference was 0.141, suggesting a smaller overall shift than in PA. However, topological differences were more pronounced, with 12 unique edges in each group’s dendrogram. This indicates substantial re-clustering of dietary behaviors associated with emotional distress.

Based on structural differences, candidate variables were selected for stepwise multiple logistic regression (MLR). Priority was given to behaviors involved in topological changes—particularly those re-clustered across groups or shifting between major branches. For PA, selected variables included the following: PA_sitting (differently positioned), PA_vigorous, and PA_leisure.time (more closely clustered in HD). For DI, key behaviors were DI_fastfood and DI_sweets (tightly clustered in HD), DI_alcoholic.drinks (shifted from peripheral to central), and DI_yellow.cheese (paired with fast food in HD). These variables were used to estimate the likelihood of HD classification, with odds ratios (ORs) quantifying the strength of each association.

A multivariate logistic regression (MLR) was conducted to examine associations between lifestyle behaviors and psychological distress, comparing low-distress (LD) and high-distress (HD) groups, with HD modeled as the outcome (HD = 1). The initial model included all candidate variables, and predictor significance was evaluated. Given high model variability and an AIC of 265.35, a stepwise approach was used to remove weaker correlates and identify the most discriminative behaviors. Final results are shown in Table 2, with odds ratios (ORs) and 95% confidence intervals. 

Vigorous physical activity (PA_vigorous) was significantly associated with higher odds of high distress; individuals reporting such activity had 1.80 times higher odds of belonging to the HD group (OR = 1.80, 95% CI: 1.33–2.50, *p* < 0.001). Similarly, frequent fast food consumption (DI_fastfood) was linked to higher distress risk (OR = 1.43, 95% CI: 1.05–1.98, *p* = 0.026). Sweets intake (DI_sweets) showed a positive trend toward higher distress, but did not reach statistical significance (OR = 1.33, 95% CI: 0.99–1.80, *p* = 0.064).

Taken together, these findings suggest that individuals with higher psychological distress tend to exhibit a complex behavioral profile—combining unhealthy dietary habits (e.g., increased fast food and sweets consumption) with compensatory behaviors like vigorous physical activity. This pattern may reflect an attempt to manage distress through mixed lifestyle strategies.

## 4. Discussion

In this work, we were interested in studying the associations between physical activity and dietary patterns in participants with different levels of emotional well-being. This study provides novel insights into the relationship between lifestyle behaviors and psychological distress in university students, revealing that individuals with high distress exhibit both distinct and more structurally consolidated patterns of physical activity and dietary intake. Multivariate and dendrogram-based analyses confirmed significant, though modest, differences in overall behavioral structure between high- and low-distress groups. Notably, emotionally distressed individuals tended to engage in compensatory lifestyle strategies, combining unhealthy dietary habits (e.g., increased fast food and sweets intake) with vigorous physical activity. These patterns—supported by both structural clustering and logistic regression results—suggest a more rigid or thematically organized lifestyle configuration under distress, possibly reflecting efforts to self-regulate through health-compromising and health-promoting behaviors in tandem.

Numerous studies have shown that frequent and strenuous physical activity positively affects a person health and well-being in general [32,33,34]. There is a correlation between regular physical activity and improvements in one’s mental well-being, physical resilience, and total subjective well-being [35,36].

Systematic review and meta-analysis of 59 studies on undergraduate students found that physical activity interventions significantly reduce symptoms of anxiety (standardized mean difference [SMD] = −0.88), depression (SMD = −0.73), and stress (SMD = −0.61). Despite considerable heterogeneity and some methodological limitations, the evidence supports physical activity as an effective means to enhance mental health in this population. However, more rigorously designed interventions are needed for stronger conclusions [37].

A second meta-analysis published in 2024 showed that moderate and vigorous physical activity to replace or intermittent sedentary behavior (SB), which could effectively prevent or improve the harm of SB to physical and mental health [38]. Overall, meta-analytic evidence robustly supports physical activity as a valuable strategy for enhancing mental health and well-being across the lifespan and in various clinical and non-clinical populations [39,40].

The regulatory benefits of PA in mood may be related to the hippocampus and neurotransmitters because the hippocampus is the central brain region that regulates anxiety, and PA could effectively promote the growth of the hippocampus nerve [41]. Animal experiments have shown that hippocampus BDNF mRNA level increases and anxious behavioral experiments was significantly reduced when mice wheel exercise [42] and regular PA could also increase the release of endorphin, thereby reducing depression, anxiety, and other negative emotion. Brain-derived neurotrophic factor (BDNF) may play an important role in this, as it is crucial for neurogenesis and cognitive function. Lower physical activity may reduce BDNF concentrations, potentially affecting mood and emotional regulation [43,44].

The results of studies conducted in recent years suggest that diet plays a key role in mental health [45,46].

Adherence to healthy or Mediterranean dietary patterns—high intake of fruits, vegetables, nuts and legumes; moderate consumption of poultry, eggs and dairy products; and only occasional consumption of red meat—has been shown to be associated with a reduced risk of depression. A systematic review and meta-analysis of observational studies by Lassale et al., 2019 [45], found that pooled estimates from four longitudinal studies show that people in the highest category of adherence to the Mediterranean diet have a lower odds/risk of depressive outcomes, with an overall estimate of 0.67 (95% CI 0.55–0.82) compared with those with the lowest adherence [47]. The authors note that the nature of these relationships is complicated by a clear trend of reverse causality between diet and mental health. For example, changes in dietary choices or preferences in response to our momentary mental state, such as “mood-enhancing foods” during periods of bad mood or changes in appetite due to stress, are common human experiences. In systematic review and meta-analysis conducted by Tavakoly in 2025, it was shown that the Mediterranean diet appears to have no potential impact on major depressive disorder. The authors suggest that this finding should be interpreted with caution and that further research on dietary patterns and depressive disorders is needed to reach more definitive conclusions [48].

The relationship between diet and mental status is undoubtedly complex and multidirectional. An increased intake of fruit, vegetables, and nuts, as well as a re-education of the intake of pro-inflammatory foods such as processed meat and trans fats, and alcohol, can reduce inflammation in the body. The consumption of foods with antioxidant and anti-inflammatory properties may provide important protection and reduce neuronal damage caused by oxidative stress [49]. Inflammation can affect the brain through active transport of cytokines across the brain endothelium or activation of vagus nerve fibers, and play a role in emotion regulation through mechanisms involving neurotransmitters including serotonin, dopamine, and norepinephrine [50]. In addition, healthy eating habits influence the proper functioning of the microbiome–gut–brain axis [51]. A healthy gut microbiome, supported by a diet rich in fiber from plant sources, can influence mood through the gut–brain axis. Dysfunction of this axis caused by poor dietary habits can lead to increased levels of emotional distress and depressive episodes. High-quality plant-based diets are typically rich in essential nutrients such as vitamins, minerals, and antioxidants that are beneficial for brain health. These nutrients can help reduce inflammation and oxidative stress, which are linked to mood disorders [46].

Unhealthy foods, sugary desserts and drinks, and low intake of fruit, vegetables, and whole-grain products are associated with elevated levels of inflammatory markers in the blood [52]. Higher levels of inflammatory biomarkers may impair neurogenesis and neuroplasticity [53,54] or the function of serotonin, dopamine, and brain-derived neurotrophic factor [55,56]. In support of this mechanism, many studies have shown a direct link between depression and prolonged psychological stress and inflammatory pathways in the brain [57]. In addition, most unhealthy plant foods have a high glycemic index (GI) and glycemic load (GL), which may affect mental health through lower content of essential nutrients for mental health or their detrimental effects on the gut microbiota and inflammatory processes [49,58,59].

A study of Iranian students found that a “Western diet” dietary pattern was not associated with mental health problems, strong inverse association was observed between the “plant-based” dietary pattern and depression [60]. Other studies have shown an inverse relationship between healthy eating patterns and severity of anxiety disorders. Higher adherence to Western and mixed dietary patterns resulted in higher anxiety disorders [61]. It was found that women who followed a healthier diet were less likely to experience mood swings and less likely to feel triggered in a social context. This group did not tend to overeat, gain weight, or overeat [62].

Participation in physical activity is also an important factor for good health behavior. The group of students studied showed high and medium levels of physical activity resulting, among other things, from participation in sports activities implemented as part of the study program. Physical activity, as emphasized by other authors, is an important mediator that influences both nutritional choices and improves mental health [63]. Therefore, our study did not show a high percentage of people with poor eating habits and low mental well-being. It appears that education combining dietary intervention and physical activity will be more effective in improving the mental state of young people.

Although females were overrepresented in the high-distress group, subgroup analyses stratified by both sex and stress level were not feasible due to small sample sizes, especially among high-distress males. We addressed this limitation by standardizing continuous variables and analyzing the full cohort as a whole. This approach allowed us to examine general behavioral and emotional patterns across adolescents while minimizing the influence of unequal subgroup sizes. Nevertheless, we acknowledge that the gender imbalance may still act as a potential confounder and recommend future studies with larger and more balanced samples.

Strengths and limitations. This study has few strengths. This case–control study is uniquely involving physically actively males and females examined to the relation between prominent eating patterns and the severity of emotional well-being through depression, anxiety, and stress levels with a mediation role of body composition and odds of the low emotional well-being. Nevertheless, a few limitations also need to be considered. One of the limitations of the present study was the cross-sectional design, which prohibits causal inference. The results of our study cannot be generalized to the population. The study group consisted of students at the University of Public Health, who have a higher physical activity level than the Polish population of their age, in addition to having a greater number of classes in the study program on the subject of good nutritional habits and physical activity. In addition, the unequal gender distribution across distress groups—particularly the small number of males in the high-distress category—prevented statistically reliable subgroup analyses stratified by both sex and psychological distress level. Consequently, we analyzed the full sample as a unified cohort. To minimize potential confounding effects related to gender and individual variability, all continuous variables were standardized prior to analysis. This normalization-based approach, commonly used in anthropometric and developmental physiology research, allowed us to focus on relative behavioral structures across the adolescent population. Another limitation was the small number of participants covering only the minimum requirements of exploratory methods. Moreover, dropout observed during recruitment lowered the sample size in comparison to the needed. Also slightly bias toward a greater number of females was a certain limitation. Additionally, first-year university students undergo numerous lifestyle transitions, including changes in diet, physical activity, and stress levels. Greater autonomy in food choices, exposure to new physical activity options, peer influence, and adaptation to a new academic environment may have affected participants’ behaviors and psychological responses. Since the data collection took place a few weeks after the beginning of the academic year, future studies should consider longitudinal approaches to monitor how these behaviors evolve over time. Such designs could help identify whether initial changes persist, stabilize, or intensify during the academic year.

In the future, more precise research tools for both eating habits and levels of physical activity and psychological well-being should also be used for this group of subjects. Comparative studies should also have been carried out in overweight and obese people with low levels of physical activity and less knowledge of proper eating habits. Additional factors worth addressing in future research include body image, dieting behaviors, participation in sport, and access to healthier food and exercise opportunities, all of which may influence student well-being during their transition to university life.

## 5. Conclusions

This study demonstrates that emotional distress in university students is linked to distinct lifestyle behavior profiles, characterized by both unhealthy eating patterns and elevated levels of vigorous physical activity. Multivariate analyses revealed meaningful differences in the structure and co-occurrence of physical activity and dietary behaviors between low- and high-distress groups. Individuals with high distress showed more tightly coupled, potentially compensatory behavior patterns, suggesting that emotional strain may drive more habitual or reactive lifestyle strategies.

These findings highlight the need for targeted interventions that address both domains of behavior simultaneously. Programs aimed at student well-being should not only promote healthy eating and physical activity separately but should also recognize the complexity of how these behaviors cluster in response to psychological distress. Screening tools that identify individuals with mixed behavior profiles—such as high exercise levels combined with high processed food intake—may help flag those at an elevated risk of emotional strain.

We recommend integrating mental health screening into campus health promotion efforts, especially for physically active students. Additionally, interventions should account for compensatory behavior patterns, recognizing that health-promoting and risk behaviors may co-occur. Finally, using cluster-based or pattern-recognition approaches—rather than focusing on isolated habits—can help better tailor behavioral interventions.

## Figures and Tables

**Table 1:** Baseline characteristics of the participants according to emotional well-being (prior to calculation data have been transformed and scaled). T-values and *p*-values are derived from t-Student test for independent groups (significant differences are shown with bold font).

		Low Distress (*n* = 130)				High Distress (*n* = 79)					
Variable		Mean	95%CI		SD	Mean	95%CI		SD	t	*p*
			Lower	Upper			Lower	Upper			
Dass21	Dass21 overall	−0.61	−0.73	−0.50	0.68	1.01	0.90	1.12	0.49	**−18.55**	**0.000**
	Depression	−0.54	−0.67	−0.42	0.71	0.90	0.73	1.06	0.73	**−14.12**	**0.000**
	Anxiety	−0.59	−0.69	−0.49	0.58	0.98	0.81	1.14	0.75	**−17.00**	**0.000**
	Stress	−0.53	−0.67	−0.40	0.77	0.88	0.73	1.03	0.66	**−13.59**	**0.000**
IPAQ	IPAQ overall	0.01	−0.17	0.18	0.99	−0.00	−0.23	0.22	1.01	0.06	0.952
	Walking intensity	0.09	−0.08	0.27	1.00	−0.16	−0.38	0.07	1.00	1.77	0.079
	Moderate intensity	0.17	−0.00	0.35	1.03	−0.28	−0.48	−0.08	0.89	**3.24**	**0.001**
	Vigorous intensity	−0.21	−0.39	−0.04	0.98	0.36	0.15	0.56	0.91	**−4.18**	**0.000**
	Sitting average	−0.02	−0.18	0.14	0.93	0.02	−0.23	0.27	1.11	−0.29	0.773
	Working domain	0.04	−0.13	0.20	0.97	−0.03	−0.25	0.20	1.01	0.43	0.667
	Transportation domain	0.14	−0.05	0.32	1.07	−0.20	−0.39	−0.01	0.85	**2.38**	**0.018**
	Domestic and gardening domain	0.19	0.01	0.37	1.03	−0.29	−0.49	−0.10	0.87	**3.45**	**0.001**
	Leisure-time domain	−0.13	−0.29	0.04	0.93	0.21	−0.03	0.46	1.09	**−2.40**	**0.017**
QEB	Whole bread	0.11	−0.07	0.28	1.03	−0.16	−0.37	0.05	0.93	1.88	0.061
	Milk	0.03	−0.15	0.20	1.02	−0.10	−0.31	0.11	0.93	0.91	0.365
	Fermented milk	0.02	−0.14	0.19	0.96	−0.06	−0.30	0.18	1.05	0.59	0.553
	Curd cheese	−0.03	−0.19	0.14	0.96	0.04	−0.19	0.26	1.00	−0.49	0.628
	Fish	−0.05	−0.23	0.13	1.05	0.03	−0.19	0.24	0.96	−0.53	0.596
	Legumes	−0.00	−0.18	0.18	1.07	−0.04	−0.25	0.17	0.93	0.28	0.780
	Fruits	−0.04	−0.20	0.13	0.97	0.11	−0.13	0.34	1.05	−1.01	0.314
	Vegetables	0.03	−0.15	0.21	1.03	−0.06	−0.27	0.15	0.95	0.65	0.517
	Fast food	−0.12	−0.25	0.01	0.76	0.25	−0.03	0.52	1.22	**−2.68**	**0.008**
	Fried meals	0.00	−0.16	0.16	0.92	0.11	−0.15	0.36	1.13	−0.74	0.460
	Yellow cheese	−0.02	−0.20	0.15	1.00	0.05	−0.17	0.28	1.01	−0.51	0.609
	Sweets	−0.13	−0.29	0.04	0.93	0.22	−0.02	0.47	1.09	**−2.45**	**0.015**
	Canned meals	−0.12	−0.25	0.01	0.74	0.26	−0.03	0.55	1.30	**−2.69**	**0.008**
	Sweetened beverages	−0.18	−0.32	−0.03	0.83	0.29	0.02	0.56	1.21	**−3.33**	**0.001**
	Energetic drinks	−0.07	−0.25	0.11	1.03	0.09	−0.13	0.31	0.97	−1.14	0.258
	Alcoholic drinks	−0.05	−0.23	0.13	1.03	0.09	−0.12	0.30	0.93	−1.00	0.320

Dass-21—Depression Anxiety Stress Scales-21 items, IPAQ—International Physical Activity Questionnaire, QEB—Questionnaire Eating Behavior, CI—confidence interval, SD—standard deviation. Significance codes: bold font—*p* < 0.05.

**Table 2:** Multiple logistic regression predicting high-distress group membership.

	Estimate	Error	z	*p*	Odds Ratio	2.50%	97.50%
Vigorous PA	0.59	0.16	3.66	0.000	1.80	1.33	2.50
DI_fastfood	0.35	0.16	2.23	0.026	1.43	1.05	1.98
DI_sweets	0.28	0.15	1.85	0.064	1.33	0.99	1.80

Footnote. PA—physical activity, DI—dietary intake, logistic regression model predicting the likelihood of belonging to the high-distress group. CI—confidence interval.

## Data Availability

The data presented in this study are available on request from the author.
